# Increased Intake of Selected Vegetables, Herbs and Fruit may Reduce Bone Turnover in Post-Menopausal Women

**DOI:** 10.3390/nu7042499

**Published:** 2015-04-08

**Authors:** Caroline Ann Gunn, Janet Louise Weber, Anne-Thea McGill, Marlena Cathorina Kruger

**Affiliations:** 1 School of Food and Nutrition, Massey Institute of Food Science and Technology, Massey University, Private Bag 11222, Palmerston North 4442, New Zealand; E-Mails: j.l.weber@massey.ac.nz (J.L.W.); m.c.kruger@massey.ac.nz (M.C.K.); 2 Liggins Institute, University of Auckland, Private Bag 92019, Auckland 1142, New Zealand; 3 School of Population Health, University of Auckland, Auckland 1142, New Zealand; E-Mail: at.mcgill@auckland.ac.nz

**Keywords:** fruit, vegetables and herbs, bone resorption, bone formation, phytochemicals, potential renal acid load

## Abstract

Increased consumption of vegetables/herbs/fruit may reduce bone turnover and urinary calcium loss in post-menopausal women because of increased intake of polyphenols and potassium, but comparative human studies are lacking. The main aim was to compare bone turnover markers and urinary calcium excretion in two randomised groups (*n* = 50) of healthy post-menopausal women consuming ≥9 servings of different vegetables/herbs/fruit combinations (three months). Group A emphasised a generic range of vegetables/herbs/fruit, whereas Group B emphasised specific vegetables/herbs/fruit with bone resorption-inhibiting properties (Scarborough Fair Diet), with both diets controlled for potential renal acid load (PRAL). Group C consumed their usual diet. Plasma bone markers, urinary electrolytes (24 h) and estimated dietary PRAL were assessed at baseline and 12 weeks. Procollagen type I N propeptide (PINP) decreased (−3.2 μg/L, *p* < 0.01) in the B group only, as did C-terminal telopeptide of type I collagen (CTX) (−0.065 μg/L, *p* < 0.01) in women with osteopenia compared to those with normal bone mineral density (BMD) within this group. Intervention Groups A and B had decreased PRAL, increased urine pH and significantly decreased urinary calcium loss. Urinary potassium increased in all groups, reflecting a dietary change. In conclusion, Group B demonstrated positive changes in both turnover markers and calcium conservation.

## 1. Introduction

Loss of bone mass leading to bone fragility, disability and fracture risk in the elderly is a public health issue due to increased life expectancy paralleling rapid ageing of the world’s population [1,2,3]. Women are vulnerable to increased bone loss during and after menopause. Maintenance of bone health with ageing is attributed to genetics, sun exposure (maintaining vitamin D levels), exercise and diet. Diets rich in vegetables and fruit [4] are important for bone health through provision of nutrients: potassium [5], phytochemicals, such as polyphenols [6] and fibre [7], and decreased sodium intake [8]. Increased consumption of vegetables and fruit provides a favourable ratio of sodium/potassium and reduces dietary acidity [9], which alleviates associated hypercalciuria [10]. Increased phytochemical intake may suppress the pro-inflammatory milieu and bone loss associated with ageing [11]. The significant association between better bone health and higher consumption of fruit and vegetables was noted in the literature over forty years ago. Researchers suggested a means of decreasing the attrition rate of bone was to “emphasise fruit and vegetables, vegetable protein and moderate amounts of milk” in the diet [12]. Population-based studies have also concluded that fruit and vegetable intake is associated with bone health [13,14,15]. The acid-base hypothesis suggests that the metabolic breakdown products from Western diets (high in grains/protein, low in fruit/vegetables) contribute to increased bone loss, as calcium is titrated to balance the metabolic acidosis [16]. Age compounds the effect due to a decline in renal function [17,18]. Few intervention studies have examined the effect on bone health of increased fruit and vegetable intake [19] or in conjunction with reduced sodium intake [20,21], whereas several studies used alkaline supplements, in place of fruit and vegetables, to determine the effects on calcium metabolism and bone markers [1,17,22]. While it has been acknowledged for over a decade that plant phytochemicals modulate bone metabolism [6,23], greater understanding has emerged of the mechanisms at the molecular level. The seminal work of Mühlbauer [24] determined that bone resorption was reduced in animals fed particular vegetables/herbs/fruit with bone resorption inhibiting properties (BRIPs) due to pharmacologically-active phytochemicals rather than their base excess [23,25,26]. Since then, the effect of phytochemicals in dried plums [27,28] and onions [23,29] on osteoclast inhibition, reduced bone loss and increased bone mineral density (BMD) [30] has been affirmed.

Recently, research has focused on a wider array of phytochemicals in plants and how small doses of specific plant phytochemicals directly suppress the inflammatory response by activating cell signalling pathways [31], enzyme production [32] or directing the differentiation of osteoblasts and osteoclasts [33]. The flavonoid hesperidin (citrus fruit) [34] regulates osteoblast differentiation, while quercetin (dried plums) and kaempferol (onions, broccoli) inhibit osteoclastic resorption, with kaempferol being particularly inhibitory. Although there are interwoven connections and multiple signalling cascades, inflammation and oxidative stress with the suppression of transcription factors nuclear factor kappa B (NFκB) and activator protein-1 (AP-1) [33] and regulator of cellular responses to oxidative stress, nuclear factor erythroid derived 2-related factor-2 (Nrf2) [35], respectively, are strongly associated with osteoclasts number and activity.

Previous studies [19,20,21] investigating a dietary approach to reducing bone loss have not included herbs, and some had minimal increases in vegetable intake compared with fruit. What is now needed is a study designed to detect any effect of specific vegetables/herbs and fruit with bone resorption inhibiting properties, on bone turnover [24], as well as to investigate calcium excretion in a free-living population of healthy postmenopausal (PM) women. We hypothesised that any change in bone resorption would be more evident in women with increased bone loss (osteopenia) compared to those with normal BMD. This study’s main aim was to compare two different diets, both high in vegetables/herbs and fruit, therefore both low in estimated dietary potential renal acid load (PRAL), on bone turnover and calcium excretion in post-menopausal women with variable bone loss. Group B was strongly encouraged to consume a combination of vegetables/herbs and fruit with known effects on bone turnover (Scarborough Fair Diet). Group A was to consume the same quantities of vegetables/herbs and fruit (nine servings/day) with no known effect on bone turnover markers, and a non-randomised group (C) of participants continued with their usual diet. The secondary aims were to compare: (1) nutrient intakes, bone markers and urinary calcium loss in the two dietary intervention groups with a control group diet; and (2) whether bone resorption markers differed in women with increased bone loss (osteopenia) compared to those with normal bone mineral density.

## 2. Experimental Section

### 2.1. Study Design

**Figure 1:**
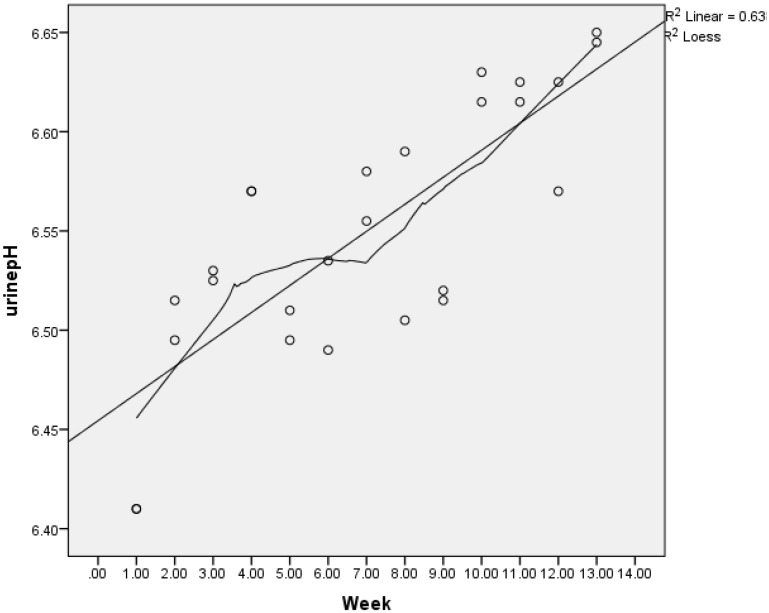
Change in urine pH in intervention Groups A and B. Urine pH was second void, fasted and self-reported by intervention participant’s twice weekly using pH dipsticks. Circles are weekly averages of groups (A and B) together. The graph is fitted with a Lowess smoother line demonstrating increasingly alkaline pH.

A three-month randomised active comparator intervention evaluated the effects of consuming ≥9 servings of vegetables and fruit/day plus selected culinary herbs on two groups of PM women emphasising differing vegetables/herbs/fruit and a third non-randomised negative control group (usual diet) as a comparison group for the two intervention groups (refer to Figure 1). Outcome measures included plasma bone turnover markers: *C*-terminal telopeptide of type I collagen (CTX) and procollagen type I N propeptide (P1NP), urinary mineral excretion, urinary pH and dietary intake, including estimated dietary potential renal acid load (PRAL).

### 2.2. Participants

One hundred healthy women (non-smokers) aged between 50 and 70 years were recruited from three New Zealand regions and randomly assigned to Groups A and B (block randomisation technique). The inclusion criteria were: at least 5 years PM, non-smoking, not on medication affecting bone and inflammatory markers, e.g., hormone replacement therapy (HRT), within the last 2 years, proton pump inhibitors or non-steroidal anti-inflammatory medication, calcium or dietary supplements. Exercise levels were recorded at baseline (New Zealand Physical Activity Questionnaire). We separately recruited 50 PM women using the same inclusion and exclusion criteria to serve as a negative control group. Separate recruitment was deemed necessary because of the significant behaviour change required in this study. Participants would have to be ready and motivated to make the dietary and behaviour changes to meet the study requirements. If they were then randomised to a control group, they may have been disappointed and possibly still increased their intake of vegetables/herbs and fruit [36]. The women were predominantly white (98%). Ethical approval was obtained from Massey University Human Research Ethics Committee (Southern A), Reference Number 11/11. All participants were fully informed of the study requirements and gave written consent. The trial was registered with the Australian and New Zealand Clinical Trials Registry (ANZCTR); trial registration: ACTRN 12611000763943.

### 2.3. Study Diets

Both intervention groups were to consume ≥9 servings of fruit and vegetables. This was composed of 6 servings of vegetables, including ≥2 servings/day of green leafy vegetables to control for vitamin K intake, 2 servings/day dairy (or calcium enriched milk substitute) to control for calcium intake and ≥1 serving of a culinary herb daily (dried/fresh) and to refrain from consuming the alternate group’s selection of vegetables/herbs and fruit. At least half of total daily vegetable servings were to include prescribed vegetables, while fruit intake was limited (≤3 servings/day) to avoid increasing fruit rather than vegetable intake [19]. To optimise reaching a physiological dose of bone modulating phytochemicals, the vegetables/herbs and fruit with bone modulating effects demonstrated in animals [24] were prescribed for one group (B) only and termed the Scarborough Fair Diet [36]. All additional vegetables/herbs and fruit required for the study were not supplied, but required to be self-funded and sourced. The controls were advised to continue eating their usual diet.

Prescribed vegetables/herbs and fruit for each group are included below (Table 1). Although there was some choice in the vegetable/fruit options, a representative sample diet was used for gross estimation of phytochemical variety in the diet. The table demonstrates that the total number of phytochemical groups present in the list of vegetables (12 vegetables and additional herbs) was higher in Group B (97) compared to Group A (77) (Supplementary Material). Group B (Scarborough Fair Diet) emphasised vegetables/herbs and fruit with proven anti-resorption inhibiting properties [24]; however, much of this range of food contains phytochemicals that have not as yet been identified as active principles.

Mühlbauer and co-workers [37] determined that a minimum effective dosage of bone resorption inhibiting fruit/vegetables (BRIFs) would be 6.2 grams of fresh fruit/vegetables per kilogram of body weight, which corresponded to food intake of BRIFs of 170 mg/per day in rats. The recommended serving numbers of BRIFs was calculated for an average 65 kilogram (kg) woman (65 × 6.2 g = ~ 400 g) of BRIFs. Assuming a serving size of 80 g, five servings/day of BRIFs were estimated to be required to demonstrate an effect on bone resorption markers. Group B had 4–6 servings/day of BRIFs specified, which was composed of 3–4 servings of BRIF vegetables and 1–2 servings of BRIF fruit. Herbs were additional to all vegetable servings for both groups due to herb intakes being in relatively small quantities (≤teaspoon). Although the aromatic phytochemicals are very concentrated [38], they could not replace vegetable servings, as this may have influenced nutrient intakes, dietary PRAL and, therefore, possibly, urinary calcium excretion.

Intervention groups were blinded to which vegetables/herbs/fruit group had proven bone modulating effects. The women were advised that this intervention was not a weight loss strategy and to maintain their normal exercise levels. Significant changes in health/medication were to be recorded bi-weekly in a diary along with urine pH (second void, fasting) and vegetable/herbs and fruit intake [36]. Participants received standardised fortnightly emails and could email queries to the study coordinator. The control group participants received instruction to maintain their normal diet, but were aware their purpose was for comparison with two intervention groups increasing vegetable and fruit intake.

### 2.4. Dietary Analysis

Three-day diet diaries (3DDDs) were completed at baseline and the end of study (Week 12). Participants recorded all food and beverages consumed over 2 weekdays and 1 weekend day, including types, brands and amounts (cups, tablespoons, *etc*.) of foods, as well as recipes for homemade dishes. Participants supplied nutritional information panels from processed food packets. All 3DDDs were checked for accuracy and completeness by a New Zealand (NZ) registered nutritionist at the first visit. Prompting methods were used for incomplete quantities or to ascertain specific food types. Data were entered into Foodworks (Version 2009, NZ, Xyris software), then transferred to the Statistical Package for Social Sciences (SPSS) (SPSS Inc. Chicago, IL, USA) (Version 20) via Microsoft Access (2007) and Excel (2007). Estimated dietary PRAL was expressed in milliequivalents per day (mEq/day) = 0.49 protein (g/day) + 0.037 phosphorus (mg/day) − 0.021 potassium (mg/day) − 0.026 magnesium (mg/day) − 0.013 calcium (mg/day) [39]. Nutrients were energy adjusted using the nutrient energy model [40].

**Table 1:** Intervention and control group diets.

**Group A fruit/vegetable/herb dietary recommendations**						
****	Herbs ^1^	Fruit ^1^			Vegetables ^1^ Green leafy	At least 5 servings/day of any vegetable (non Group B); examples of commonly-consumed vegetables
****	Basil, mint, oregano	Apple	Banana	Other fruit (not citrus or prunes)	Spinach, silver beet, white/green cabbage	Carrot, pumpkin, peas, cauliflower, courgettes,
**No. of servings/day**	1 culinary serving	1	1	1	1	
**Group B fruit/vegetable/herb dietary recommendations**						
****	Herbs	Fruit			Vegetables Green leafy	Other vegetables (≥2–3 servings from this category) and ≤2 servings self-selected
**No. of servings**	Parsley sage, rosemary, thyme, garlic	Prunes	Oranges/other citrus	Other fruit (not banana or apple)	Chinese cabbage, e.g., bok choy, red cabbage, lettuce, rocket	Onions, broccoli, tomatoes, mushrooms, cucumber, leeks, green beans
	1 culinary serving	1	1	1	1	
**Group C dietary recommendations—Continue with usual diet (no change)**						

^1^ All serving sizes according to New Zealand Ministry of Health guidelines: fruit/vegetables (F/V) = 50–80 g or 0.5 cup cooked or 1 cup raw (salad greens) or 1 medium fruit, starchy vegetables (135 g); protein includes meat, fish, eggs, nuts/seeds and legumes; herbs are additional to 9 servings of F/V and in teaspoon quantities.

### 2.5. Specimen Collection and Analysis

Baseline and end of study plasma markers included: bone markers of formation, procollagen type I N propeptide (P1NP), and resorption, C-terminal telopeptide of type I collagen (CTX). In short, overnight fasted blood samples were taken between 8–10 am, centrifuged (3000 revolutions per minute (rpm), separated and stored (−80 °C)) until bone markers were analysed at Canterbury Health Endocrine Laboratory, Christchurch, NZ (Roche Elecsys 2010, Roche Diagnostics) [36]. Twenty-four-hour urine samples were collected, measured and frozen until samples were analysed by atomic absorption flame emission spectrophotometry [36].

### 2.6. Bone Densitometry

Dual X-ray absorptiometry (DXA) scans of lumbar spine (L1–L4) and hip (total and femoral neck) was performed using a Hologic QDR-Discovery A densitometer (Hologic Inc., Bedford, MA, USA) giving measures of bone mineral content (BMC) (grams), BMD (grams/centimetre^2^), T and Z scores, body fat and lean measures (android and gynoid). The machines were calibrated daily with an *in vivo* reproducibility of coefficient of variation 0.45%–0.54% for all measured sites. BMD was determined at baseline, and the results were classified according to the World Health Organisation (WHO) classification [41,42]. For comparison between groups, femoral neck T scores were used rather than lumbar spine or total hip [43].

### 2.7. Statistical Analyses

SPSS (Version 20) was used for all analyses. Data were checked for the distribution using the Kolmogorov–Smirnov statistical test and Levene’s test for equality of variance. Parametric statistical analysis was performed for normally distributed data; otherwise non-parametric tests (Kruskal-Wallis test) were used (potassium urinary excretion) or logarithmic transformations applied prior to one-way ANOVA. ANOVA was used to compare bone and urinary mineral excretion data. *Post hoc* tests (Scheffe and Tukey-B) were used to determine group difference, as well as Student *t*-tests were used for within-group differences. Two-way ANOVA was used to determine interaction effects between diet and BMD groups on bone markers using multivariate analysis of variance (MANOVA). To avoid type 1 errors due to multiple comparisons being made with the pairwise comparison of the levels of each factor (BMD) within the levels of the other factor (group), the Sidak correction was applied. Repeated-measures ANOVAs were used to determine differences between intervention groups using baseline CTX as the covariate. Most data are expressed as means and standard deviations (SD) or 95% confidence intervals. The effect size was calculated for the change in bone markers CTX and P1NP for each group using standard deviations of either the intervention or control group and reporting the more conservative effect size. The number of subjects required to demonstrate a reduction in resorption marker CTX was calculated to be 32 (minimum) in each group, and this was determined using a power calculation based on demonstrating a difference of ~8% in the primary outcome variable (CTX) with 80% power and alpha of 0.05 (2-sided test) and accepting 0.4 μg/mL as the mean CTX of this population (26). To detect any differences between the 2 diets and allowing for withdrawals, non-compliance or maintenance (~25%), a sample size of approximately 50 women were needed in each group.

## 3. Results

### 3.1. Compliance

Bi-weekly diaries kept by intervention group participants (A and B) indicate compliance with dietary counselling to increase the consumption of vegetables/herbs/fruit, and this was confirmed with increases in urinary potassium excretion. The control group received no dietary counselling to increase fruit/vegetables intake, but also had increased urinary potassium excretion (Table 2).

**Table 2:** Changes in urinary mineral excretion in the three study groups (A, B, C) from baseline.

Urinary Mineral Excretion mmol/24 h	*n*	Baseline	*n*	End	Change	% Change	*p* Change ^1^ <
Calcium A	29	3.9 ± 2.0^2^	29	2.7 ± 1.5 ^2^	−1.2 (−1.8–−0.5) ^3^	−26	0.002
Calcium B	36	4.8 ± 2.1	36	3.5 ± 1.8	−1.3 (−2.0–−0.6)	−24	0.001
Calcium C	22	4.2 ± 1.6	22	3.6 ± 1.6	−0.61 (−1.6–−0.2)	2	0.14
*p* change between groups					N/S	<0.05	
Potassium A	29	135 ± 91	29	221 ± 199	86 (−5–167)	93	0.04
Potassium B	36	174 ± 189	36	260 ± 217	85 (−9–178)	141	0.07
Potassium C	22	187 ± 150	22	291 ± 265	104 (−27–235)	93	0.10
*p* change between groups					NS	NS	
Sodium A	29	256 ± 119	29	90 ± 50	−164 (−117–−212)	−48	0.001
Sodium B	36	274 ± 114	36	110 ± 64	−166 (131–−202)	−50	0.001
Sodium C	22	260 ± 108	22	103 ± 54	−166 (−120–−213)	−49	0.02
*p* change between groups					NS	NS	
Magnesium A	29	4.0 ± 1.9	29	3.9 ± 1.4	−0.04 (−0.8–0.8)	−28	0.9
Magnesium B	36	4.7 ± 1.6	36	4.4 ± 2.3	−0.08 (−0.9–0.7)	−5	0.9
Magnesium C	22	4.1 ± 1.5	22	4.3 ± 1.5	0.1 (−0.6–0.8)	10	0.8
*p* change between groups					NS	NS	
Creatinine A	29	8929 ± 3191	29	9881 ± 2999	952 (−659–2563)	N/A	0.2
Creatinine B	36	9017 ± 2591	36	8344 ± 3279	−481 (−1992–1029)	N/A	0.5
Creatinine C	22	9169 ± 2602	22	10,069 ± 2768	899 (−717–2516)	N/A	0.3
*p* change between groups					NS		

^1^ Significance of change between groups determined by ANOVA, except potassium (Kruskal-Wallis); within-group change was determined by Student *t*-test; ^2^ baseline and end values show means ± SDs; ^3^ all change values are means (95% CIs).

### 3.2. Anthropometric and Bone Mineral Density Measurements

Baseline measurements showed that intervention groups were similar in age, BMD and most anthropometric measures. (Table 3). There was no change in weight, BMI or blood pressure during the study. The mean age of intervention group (A, B) women was 60 years (standard deviation: 4.2, 4.2) and 11 years since menopause (5.1, 4.2). BMD was determined at baseline, and the results were classified according to WHO classification [41,42]. Most women in the intervention groups had osteopenia (52%, 52%) while more in Group A had normal BMD (40%, 32%) and more in Group B had osteoporosis (8%, 16%) (previously unaware) [43]. The control group was matched with the intervention groups for most measures; however, BMI was slightly lower in control Group C compared to the intervention groups.

**Table 3:** Anthropometric and bone mineral density (BMD) characteristics of the study population.

Characteristics ^1^	Group A *n* = 49	Group B *n* = 50	Group C *n* = 43
Age (years) ^2^	60 ± 4 (53–68,60) ^2^	60 ± 4 (51–71,61)	61 ± 5 (51–71,61)
YSM ^3^	11 ± 5 (9–12)	11 ± 4 (9–12)	11 ± 5 (9–12)
Weight (kg) (baseline)	72 ± 13 (68–76)	70 ± 13 (66–73)	66 ± 11 (63–70)
Weight (kg) (end)	72 ± 12.5 (68–74)	70 ± 13 (66–73)	66 ± 11 (62–70)
Height (metres)	1.6 ± .5 (1.61–1.64)	1.65 ± 7 (1.62–1.167)	1.63 ± 7 (1.61–1.66)
BMI ^4^ (kg/m^2^)	27 ± 5 (26–29)	25 ± 5 (24–27)	24 ± 5 (23–26)
Body fat %	40 ± 6.6 (38–42)	38 ± 7 (36–40)	37 ± 6 (35–39)
BP ^5^ systolic (mmHg) base	131 ± 16 (126–135)	132 ± 18 (127–137)	129 ± 18 (124–135)
BP systolic (mmHg) end	131 ± 15 (126–135)	129 ± 17 (125–134)	127 ± 18 (122–133)
BP diastolic(mmHg) base	80 ± 10 (77–83)	79 ± 10 (76–81)	78 ± 9 (75–81)
BP diastolic (mmHg) end	79 ± 10 (76–82)	79 ± 10 (76–82)	78 ± 9 (75–80)
BMD ^6^ (*n* = 142)			
Normal BMD (%)	20 (40)	16 (32)	15 (36)
Osteopenia (%)	26 (52)	26 (52)	22 (54)
Osteoporotic (%)	4 (8)	8(16)	4 (10)
Spine BMC (g/cm)	53 ± 13 (49–57)	55 ± 10 (52–58)	55 ± 13 (50–59)
Spine BMD (g/cm^2^)	0.95 ± 15 (0.90–0.99)	0.94 ± 14 (0.90–0.98)	0.97 ± 0.20 (0.90–1.00)
Spine *t*-score	−0.8 ± 1.2 (−1.1–−0.5)	−0.9 ± 1.3 (−1.3–−0.6)	−0.6 ± 1.6 (−1.1–−0.1)
Hip BMC (g/cm)	30 ± 5 (29–32)	29 ± 5 (28–31)	30 ± 5 (29–32)
Hip BMD (g/cm^2^)	0.88 ±11 (0.85–0.91)	0.86 ± 0.14 (0.82–0.89)	0.88 ± 0.01 (0.84–0.92)
Hip *t*-score	−0.5 ± 0.9 (−0.8–−0.3)	−0.69 ± 1.1 (−1.0–−0.3)	−0.48 ± 1.0 (−0.8–−0.2)
Femoral neck hip BMC (g/cm)	3.7 ±0.7 (3.5–3.9)	3.5 ± 0.8 (3.3–3.8)	3.8 ± 0.6 (3.6–4.0)
Femoral neck hip BMD (g/cm^2^)	0.76 ± 0.12 (0.72–0.79)	0.72 ± 0.11 (0.69–0.75)	0.75 ± 0.12 (0.72–0.79)
Femoral neck hip *t*-score	−0.8 ± 1.0 (−1.2–−0.5)	−1.2 ± 1.0 (−1.5–−0.9)	−0.9 ± 1.1 (−1.2–−0.5)

^1^ One-way ANOVA, *p* < 0.05 considered significant between groups; values are mean ± SD (95% CI), except for age ^2^, which is mean ± SD (range, median); ^3^ YSM = years since menopause; ^4^ Body Mass Index, ^5^ BP = blood pressure; ^6^ Bone mineral density; normal: BMD higher than 1 SD below young adult female reference mean (*t*-score ≥ −1 SD); osteopenia: BMD 1 SD or more below the young female adult mean (−1 < *t*-score < −2.5 SD); osteoporosis: BMD 2.5 SD or more below the young female adult mean (*t*-score ≤ −2.5 SD).

### 3.3. Changes in Food Group Servings

At baseline, there was no difference between the intervention groups or control group in the number of servings of food/day. The main changes reported in serving numbers were in the vegetables/fruit and breads/cereals food groups, where intervention groups differed significantly from the control group (Table 4). Both intervention Groups A and B increased their fruit servings/day by 0.9 (A) and 1.3 (B) and vegetable servings/day by 2.0 (A) and 2.8 (B), while the control group did not. Breads/cereals servings/day decreased in the intervention groups by 1.4 (A) and 1.2 (B) compared to no change for the control group (C). The intake of dairy servings/day decreased minimally in Group A (−0.2, *p* < 0.03) compared to no change in Group B (*p* < 0.05), while control Group C, it was reduced (−0.4, *p* < 0.003). No change was reported in the intervention groups for the meat/protein food group, and this was also unchanged in control Group C. The increased servings in fruit/vegetable and decrease in breads/cereals were significantly related to decreased dietary PRAL [44] in the intervention groups (−17, −22 mEq/day) compared to no change in the control group (*p* < 0.001).

**Table 4:** Changes in food group servings and estimated dietary PRAL from baseline to the end of the dietary intervention.

Servings ^1^ /Day	Group A *n* = 47	Group B *n* = 50	Group C (Control) *n* = 41	*p* ^2^
Fruit (baseline) ^3^	2.0 ± 0.9 (0–3.8)	1.8 ± 0 .9 (0.3–3.8)	2.2 ± 1.2 (0–4.7)	0.15
Fruit (final)	2.9 ± 1.4 (0.50–7.0)	3.0 ± 1.0 (1.3–5.3)	2.2 ± 1.4 (0–6.3)	0.003
Change ^4^	0.9 ± 1.5 (−2.3–4.8)	1.3 ± 1.1 (−0.8–4.0)	−0.06 ± 1.0 (−2.0–2.0)	0.001
	*P* < 0.001	*p* < 0.001	*p* = 0.51	
Vegetables (baseline)	3.4 ± 1.2 (1.3–6.0)	3.3 ± 1.3 (1.0–7.8)	3.7 ± 1.3 (1.2–7.0)	0.21
Vegetables(end)	5. 5 ± 1.9 (1.7–10)	6.1 ± 1.9 (2.7–12.2)	4.0 ± 1.2 (0.7–6.3)	0.001
Change ^4^	2.0 ± 2.2 (−3.7–7.3)	2.8 ± 2.1 (−2.5–8.2)	0.2 ± 1.5 (−3.7–2.8)	0.001
	*p* < 0.001	*p* < 0.001	*p* = 0.49	
Bread/cereals (baseline)	4.5 ± 1.60 (1.0–8.3)	4.4 ± 1.4 (0.3–7.5)	4.2 ± 1.4 (1.7–7.5)	0.54
Bread/cereals (end)	3.4 ± 1.52 (0.83–7.8)	3.3 ± 1.5 (0–7.0)	4.1 ± 0.7 (0.5–8.0)	0.03
Change	−1.4 ± 1.9 (−5.7–2.3)	−1.2 ± 1.5 (−5.7–1.5)	−0.22 ± 1.5 (−4.7–2.2)	0.03
	*p* < 0.001	*p* < 0.001	*p* = 0.48	
Dairy (baseline)	1.5 ± 0.74 (0.3–2.8)	1.5 ± 0.9 (0.2–4.2)	1.8 ± 1 (0.3–5.3)	0.28
Dairy (end)	1.3 ± 1.0 (0.0–5.0)	1.5 ± 0.8 (0.2–3.5)	1.4 ± 0.0 (0.0–4.5)	0.77
Change	−0.2 ± 1.0 (−2.0–3.3)	−0.08 ± 0.8 (−2.3–1.7)	−0.4 ± 0.8 (−2.0–1.5)	0.24
	*p* < 0.03	*p* = 0.5	*p* < 0.003	
Meat and protein (baseline)	1.9 ± 0.9 (0.3–4.3)	1.8 ± 0.8 (0.3–5.0)	2.0 ± 0.8 (0.5–4.3)	0.43
Meat and protein (end)	1.8 ± 1.0 (0.2–4.8)	1.7 ± 0.8 (0.3–4.0)	2.0 ± 1.0 (0.2–6.3)	0.34
Change	−0.1 ± 1.0 (−2.7–2.5)	−0.1 ± 0.9 (−2.0–2.0)	−0.1 ± 1.0 (−2.2–3.8)	0.99
	*p* = 0.46	*p* = 0.40	*p* = 0.69	
PRAL (baseline) mEq/day ^5^	−0.1 ± 16 (−36–31)	−1.2 ± 15 (−34–26)	−1.8 ± 13 (−43–27)	0.76
PRAL (end) mEq/day	−17 ± 17 (−45–27)	−23 ± 16 (−65–10)	−3 ± 16 (−36–40)	0.001
Change ^5^	−17± 17 (−55–15)	−22 ± 25 (−65–10)	−1.6 ± 18 (−30–60)	0.001
	*p* < 0.001	*p* < 0.001	*p* = 0.57	

^1^ All serving sizes according to New Zealand Ministry of Health guidelines: fruit/vegetables = 50–80 g, or 0.5 cup cooked, or 1 cup raw (salad greens), or 1 medium fruit, starchy vegetables (135 g); protein includes meat, fish, eggs, nuts/seeds and beans; ^2^ one-way ANOVA; *p*-values < 0.05 are considered statistically significant; ^3^ values are means ± SD (minimum and maximum); ^4^ paired *t*-test showing significance in changes within each group; ^5^ PRAL, estimated dietary potential renal acid load expressed in milliequivalents per day (mEq/day) = 0.49 protein (g/day) + 0.037 phosphorus (mg/day) − 0.021 potassium (mg/day) − 0.026 magnesium (mg/day) − 0.013 calcium (mg/day).

### 3.4. Changes in Dietary Nutrient Intake

Nutrient intake changes were assessed from baseline and the end of the study using three-day diet diaries. Nutrient intake was similar for intervention groups at baseline (Table 5), and no significant differences were seen between intervention and control groups. At the end of the study, calcium, potassium, magnesium, folate and fibre intake had increased in the intervention groups. Energy intake was adjusted (8000 kJ) to more accurately reflect the nutrient intake change in the composition of the diet during the intervention [40].

**Table 5:** Nutrient intake for each group at baseline and changes at 3 months.

Nutrientse	Group A Intervention *n* = 47	Group B Intervention *n* = 50	Group C Control *n* = 41	EAR ^1^/AI ^2^ RDI ^3^/SDT ^4^	*p* ^5^
Baseline					
Protein (g) ^6^	86 ± 17 (49–128)	82 ± 18 (46–128)	83.5 ± 13.6 (56–117)	37 ^1^/46 ^3^	
Change	2.2 ± 19 (−67–35)	2.0 ± 17 (−34–45)	0.10 ± 16 (−53–35)		0.8
Fat (g)	72 ± 16 (35–98)	75 ± 14 (47–102)	74 ± 17 (42–112)	N/A	
Change	2.30 ± 15 (−36–24)	2 ± 19 (−49–57)	4 ± 20 (−69–41)		0.9
CHO (g)	220 ± 43 (150–350)	216 ± 32 (107–275)	220 ± 41 (132–304)	N/A	
Change	19 ± 72 (−115–195)	5.2 ± 52 (−148–133)	6 ± 65 (−155–124)		0.5
Fibre (g)	27 ± 8 (14–50)	27 ± 8 (12–56)	28 ± 8 (13–43)	25 ^2^/28 ^4^	
Change	5.7 ± 6.6 (−22–7)	6.4 ± 10.3 (−28–18)	−0.5 ± 0.0 (−24–24)		0.01
Folate (μg)	368 ± 153 (102–812)	380 ± 225 (151–1646)	381 ± 134 (205–687)	400 ^3^/600 ^4^	
Change	109 ± 213 (−865–447)	121 ± 250 (−561–1177)	−9 ± 16 (−237–548)		0.01
Sodium (mg)	2345 ± 618 (1261–4046)	2766 ± 900 (1242–4593)	2551 ± 979 (947–6405)	1600 ^4^	
Change	−63 ± 887 (−2466–1566)	−376 ± 943 (−1561–2461)	24.5 ± 1256 (−2147–3230)		0.2
Potassium (mg)	3695 ± 766 (2400–6261)	3643 ± 1000 (2013–6902)	3781 ± 790 (2410–6027)	2800 ^2^/4700 ^4^	
Change	935 ± 953 (−3246–775)	1393 ± 1375 (−5401–2483)	64 ± 933 (−1758–2414)		0.001
Magnesium (mg)	365 ± 930 (232–694)	366 ± 105 (208–667)	385 ± 112 (231–941)	265 ^1^/320 ^3^	
Change	73 ± 134 (−548–298)	54 ± 106 (−285–229)	−20 ± 113 (−143–532)		0.001
Calcium (mg)	850 ± 257 (375–1532)	872 ± 347 (404–2,166)	905 ± 270 (396–1632)	1100 ^1^/1300 ^3^	
Change	181 ± 353 (−935–635)	164 ± 41 (−974–1514)	5 ± 293 (−621–679)		0.03

NZ reference values are the: ^1^ estimated average requirement (EAR) (50% population requirements); and/or ^2^ adequate intake (AI); ^3^ recommended daily intake (RDI) (98% of population); and/or ^4^ “suggested dietary target” (SDT); ^5^ one-way ANOVA between groups, *p* < 0.05 was considered significant; ^6^ values are means ± standard deviation (SD) (minimum and maximum) or percentages; nutrients energy adjusted (8000 kJ).

### 3.5. Urinary pH and Vegetable/Herb/Fruit Intake

Bi-weekly diaries kept by intervention groups demonstrated a mean increase in urinary pH from 6.40 to 6.65 pH units for Group A and 6.39 to 6.65 for Group B (Figure 1) compared to no change in the control group’s baseline and ending pH (6.43, 6.44), (*p* < 0.001). Intervention groups (A, B) reported mean servings/day of green leafy vegetables (2.7, 2.3), total vegetables (6.1, 6.3) and fruit (2.9, 2.9), with no significant differences between the two groups dairy intakes; herbs, however, were reported to increase more in Group A than B (1.3, 0.91 *p* < 0.001).

### 3.6. Urinary Mineral Excretion

Due to the time and effort involved in completing two 24-h urine collections, women were advised that they only need do this if they could manage it within their work commitments; therefore, subsets of each group, A (*n* = 29), B (*n* = 36), C (*n* = 22), provided baseline/end-of-study 24-h urine samples. No significant differences in mineral excretion were noted at baseline between intervention groups or between intervention and control groups. The urinary potassium excretion increase during the study may confirm the compliance of the intervention groups with the study aims to increase vegetables and fruit, thereby increasing urinary potassium output. Group A increased by 86 mmol/day (93%, *p* < 0.04) and Group B by 85 mmol/day (141%, *p* < 0.07); however, control Group C (normal diet) increased by 104 mmol/day (93%, *p* = 0.10), with no significant difference between intervention and control groups (*p* = 0.96) (Table 5). A significant change in the percentage calcium excretion was seen in the intervention groups compared to control with calcium excretion decreased by 1.2 mmol/day in Group A (26%) and 1.3 mmol/day in Group B (24%), compared to a decrease of 0.6 mmol/day (2%) in Group C (*p* < 0.14). 

Sodium excretion decreased in the intervention groups: group A by 164 mmol/day (48%, *p* < 0.001) and group B by 166 mmol/day (50%, *p* < 0.001). The control Group C also decreased by 166 mmol/day (49 %, *p* < 0.02) with no difference between intervention and control groups.

### 3.7. Bone Turnover Markers

**Figure 2:**
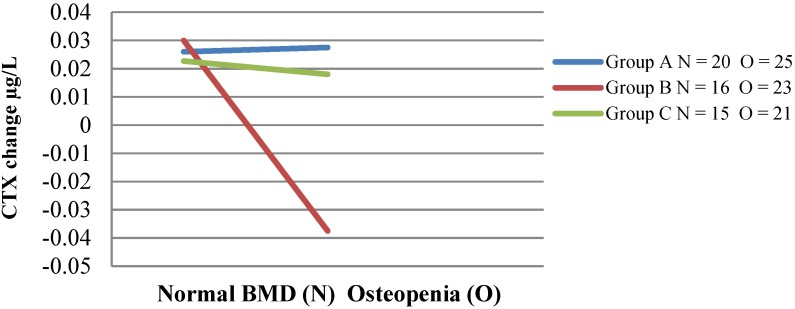
Change in CTX in women with normal bone mineral density or osteopenia. This figure demonstrates the interaction effect of group and bone mineral density status. *C*-terminal telopeptide of type I collagen (CTX) decreased significantly in the women with increased bone loss (osteopenia) in group (B) compared to the other two groups of women (A, C) with osteopenia.

Baseline bone markers of resorption (CTX) and formation (P1NP) were the same in both intervention groups, and no difference was seen between the intervention groups and the control group (Table 6). Bone formation marker P1NP decreased significantly in intervention Group B from 49.7 to 45.9 μg/L (*p* < 0.01) compared to no significant change in Group A. The effect size of the P1NP reduction between intervention groups was 0.2 (small); however, this is biologically significant. There was no change in P1NP levels in the control Group C with an effect size of 0.4 (moderate) when compared with Group B. There was no significant main effect of the intervention diet group (A, B) on change in CTX. Repeated-measures ANOVA was also performed on bone markers using age and baseline CTX as covariates. CTX decreased from 0.42 to 0.41 μg/L (*p* = 0.35) in the Scarborough Fair group (B), while CTX of Group A increased from 0.37 to 0.40 μg/L (*p* < 0.03). Although there was a decrease in CTX within the group (B), the difference was not significant, nor was any difference between intervention groups. There was, however, a significant reduction in CTX in Group B seen between those with normal bone mineral density and those with osteopenia (Figure 2). A two-way ANOVA examined the interaction effect of group and baseline BMD (normal BMD and osteopenia) on the change in CTX (due to low numbers, no comparison was available for women with osteoporosis). A significant interaction effect (*p* = 0.039) existed between the three groups (A, B, C) based on BMD. The B group women with osteopenia (*n* = 23) consuming the Scarborough Fair range of vegetables/herbs and fruit had significantly decreased levels of resorption marker CTX, with a mean difference of −0.065 μg/L, *p* < 0.01, compared to women with normal BMD, and this effect was not observed in intervention Group A (*n* = 25), where CTX levels were unchanged for women with normal BMD compared to those with osteopenia. The difference in change in CTX between intervention groups assessed by BMD corresponded to an effect size of 0.8. No significant change was seen in control Group C (*n* = 21) CTX levels, and when compared with Group B, there was an effect size of 0.4. 

**Table 6:** Changes in bone markers within each group.

Bone Markers	*n*	Intervention Group A	*n*	Intervention Group B	*n*	Non Intervention Group C	*p* between Groups ^1^
CTX baseline μg/L ^2^	48	0.37 (0.34–0.41)	50	0.42 (0.38–0.45)	43	0.39 (0.33–0.45)	0.4
CTX mid μg/L	41	0.39 (0.35–0.43)	47	0.43 (0.39–0.47)			0.2
CTX final μg/L	47	0.40 (0.36–0.44)	47	0.41 (0.38–0.45)	39	0.40 (0.35–0.46)	0.8
Change in CTX		0.03 (0.01–0.03)		−0.01 (−0.03–0.03)		0.01 (−0.01–0.03)	0.4
*p*-change		*p <* 0.03		*p* = 0.9		*p* = 0.3	
P1NP μg/L	48	44.2 (39.8–48.7)	50	49.7 (45.1–54.2)	43	45.0 (39.0–50.8)	0.2
P1NP mid μg/L	41	43.8 (38.9–48.6)	47	46.7 (42.5–50.9)			0.4
P1NP end μg/L	47	43.3 (39.3–47.3)	47	45.9 (42.4–49.5)	39	46.6 (38.5–54.6)	0.6
Change in P1NP		−0.9 (−2.4–4.3)		−3.2 (−5.9–0.4)		1.8 (−3.1–6.6)	0.2
*p*-change		*p* = 0.6		*p* < 0.01		*p* = 0.5	

^1^ *p*-values for group comparisons are derived from ANOVA; ^2^ values are means (95% CI); *p* change by Student *t*-tests. CTX, *C*-terminal telopeptide of type 1 collagen; P1NP, procollagen type 1 N propeptide.

## 4. Discussion

The main finding of our study was a decrease in bone turnover markers in Group B osteopenic women consuming the Scarborough Fair dietary mix of vegetables/herbs and fruit. Resorptive activity (CTX) in osteopenic women and formation activity (P1NP) decreased in all women in this group, reflecting lowered bone turnover. The decrease in P1NP was considered biologically significant. The decrease in CTX in Group B women with osteopenia differed significantly from intervention Group A women with osteopenia who demonstrated no change in CTX and, likewise, the control Group C. These results indicate that neither the increased dietary potassium intake nor decreased dietary PRAL in the intervention Group A was sufficient to reduce bone markers. The Scarborough Fair combination of vegetables/herbs and fruit has shown effects on resorption in the rat model [45], but not previously in humans. The antiresorptive effect was, however, evident only in women with increased bone loss (osteopenia), not women with normal BMD. This effect is therefore very useful, and appears to ameliorate a deficiency situation, whilst presumably not affecting women with normal BMD.

Significantly less (%) urinary calcium loss was seen in both intervention groups compared to the control (C). Whether the intervention groups prescriptive dietary instructions to consume more vegetables (especially leafy greens), which are more alkaline forming than fruits, or lowered dietary PRAL contributed to the reduced urinary calcium was not determined. However, only the intervention groups demonstrated lowered dietary PRAL and increased alkalinity of urinary pH (refer Figure 1), whereas the control group demonstrated only increased urinary potassium. Eighty five percent of potassium consumed in the diet is absorbed, and of that, 80%–90% is excreted in the urine [5]. Therefore, increased urinary potassium excretion reflects increased potassium intake sourced predominantly in this study from vegetables and fruit in intervention groups who received specific counselling to increase this food group [46].

The urine calcium lowering effect observed when dietary acidity decreases is considered the main reason for lower calcium excretion, rather than increased potassium intake [1,47]. Recent research has shown bicarbonate allows for increased calcium uptake in intestine, but prevents its loss in the urine [10], while others have shown when dietary PRAL is higher, there will be increased calcium lost in the urine [48]. The dietary acid load of Groups A and B was significantly reduced in this study, and calcium retention was higher compared with Group C.

The inverse relationship that exists between potassium consumption and calcium excretion has been attributed to lowered intestinal absorption of calcium. Lowered intestinal absorption of calcium was observed in participants who were deriving most of their potassium intake from dairy, meat and cereal grains, rather than vegetable and fruit sources [49], which may have been the case with Group C participants, who maintained a high potassium excretion, but had lower calcium excretion and no significant change in their dietary acid load or urine pH when compared with intervention groups. Analysis of Foodworks data from three-day diet diaries determined that our intervention groups indeed derived most of their potassium intake from vegetables and fruit food group. Sodium intake also may account for obligatory calcium loss, as both share the same reabsorption transporter in the proximal tubule [50]; however, since all groups had significant reductions in urinary sodium output, reduced intake cannot account for differences in urinary calcium excretion between groups. Reported dietary intake varied significantly between intervention and control groups at the end of study, with reductions in food group servings of breads/cereals and increases in/vegetables/fruit, resulting in significantly lower estimated dietary PRAL and sodium intake in intervention groups compared with control Group C [51]. Macronutrient intakes remained the same; however, micronutrient intakes of potassium, magnesium and calcium increased significantly in the intervention groups. An improved profile of potassium, magnesium, calcium and sodium dietary intake according to national and international nutrient reference intake values was seen in intervention groups by the end of the study. However, despite compliance with ≥9 servings of fruit/vegetable/day, inclusion of two servings of green leafy vegetables/day and a reduction in breads/cereals food group, only one intervention group (Group B) achieved national and internationally-suggested dietary targets (SDT) for potassium (4700 mg/day) [52,53], and the NZ SDT for folate (SDT 600 μg/day) was not reached by either intervention group. Although sodium intake was significantly decreased, at the study end, it remained more than the SDT (≤1600 mg/day). These results suggest that even greater change is required in current eating patterns to achieve recommended targets or targets that are unattainable for the majority [54]. It is debatable whether an RCT, while being the gold standard when using pills and placebos, is the right method for dietary change in a free-living group of people, as many different aspects of diets change when fruit and vegetable intake increase, e.g., the intake of other food groups may be lowered. Dietary interventions become complex if not done as a metabolic study with participants monitored on site in a nutrition unit. This dietary study wanted to determine if free-living women could make a sufficient dietary change to affect bone markers and calcium excretion. This dietary change was to be sustainable and not involve consumption of large quantities of a single food daily for three months, e.g., prunes, or requiring supplementation of the diet with high potency extracts or mineral supplements, such as calcium, which also lower bone resorption.

The strengths of the study are the following: randomisation to differing selections of vegetables, herbs and fruit with one combination specifically linked to bone health; that women with osteopenia only had the greatest effect of the diet to improve bone marker activity; a community-based real-life setting involving participants purchasing, storing and cooking additional vegetables, herbs and fruit, allowing generalisation of the results to similar free-living populations; control of potential confounders, such as PRAL, dietary calcium/vitamin K, by specifying minimum intakes of servings dairy/green leafy vegetables; no additional calcium/vitamin D supplementation, fortification or requirement to eat large amounts of a single food; and lifestyle factors, such as weight and levels of exercise being unchanged during this study. However a weakness in this study is that additional confounders are likely to be present in a community-based intervention and these potential confounders were not included in the analysis; therefore, they may have had some effect on the results. Another potential limitation is selection bias for healthy women who had sufficient motivation and the financial means to provide for the additional vegetables/herbs and fruit. Additionally, Group C, the controls, were not randomised and appeared to increase ‘healthy’ foods, possibly including vegetable and fruit, giving a “Hawthorne effect”. This effect is demonstrated in studies, whereby participants who sign an informed consent and understand that they are to be compared with other groups who are making a change (increasing vegetable and fruit intake) influences some in the control group to also make the (dietary) change and partially adopt a treatment group pattern [46]. This change influenced the comparison of the bone turnover, urinalysis and dietary results of the three groups and limits the generalisation of the results to healthy PM women who consume a diet high in potassium. However, it is possible that a greater difference in bone markers would be seen with other groups of women initially consuming diets lower in fruit and vegetables. This study was a pilot and, therefore, not sufficiently powered to determine a strong effect between women of different bone mineral density status and increased vegetable/herb and fruit intakes. Several study participants declined or were unavailable for DEXA scans; hence, we had only 142 women to compare BMD, rather than the whole group. Future studies should allow for sufficient participants, including a small proportion who want to be part of a dietary change study, but opt not to have a DEXA scan.

The effect of the Scarborough Fair combination of vegetables/herbs and fruit on bone turnover seen in this study would need to be confirmed by a larger study. Future trials should stratify for baseline BMD, ensuring sufficient participants with osteopenia and osteoporosis, and be sufficiently powered to allow analysis between all BMD groups. A longer study time period of at least two years would allow for bone health assessment using DEXA and/or peripheral QCT to determine if changes occurred in bone mineral density, strength or quality [55].

## 5. Conclusions

In conclusion, this is the first study showing increased intake of a selection of vegetables/herbs and fruit decreased bone formation (P1NP) and resorptive (CTX) markers (osteopenic women). Lowered dietary PRAL was associated with urinary calcium conservation in intervention Groups A and B compared to control diet Group C. Downward modulation of bone turnover markers suggests that the additive effect of active phytochemical agents present in the SF selection of vegetables/herbs and fruit reduces bone turnover, particularly in those women with osteopenia.
